# Microwave (MW), Ultrasound (US) and Combined Synergic MW-US Strategies for Rapid Functionalization of Pharmaceutical Use Phenols

**DOI:** 10.3390/molecules23092360

**Published:** 2018-09-15

**Authors:** Anna Pawełczyk, Katarzyna Sowa-Kasprzak, Dorota Olender, Lucjusz Zaprutko

**Affiliations:** Department of Organic Chemistry, Pharmaceutical Faculty, Poznan University of Medical Sciences, Grunwaldzka 6, 60-780 Poznań, Poland; kasik@ump.edu.pl (K.S.-K.); dolender@ump.edu.pl (D.O.); zaprutko@ump.edu.pl (L.Z.)

**Keywords:** phenol, linker, etherification, MW/US strategy, microwave irradiation, ultrasound, green synthesis, “Lego” chemistry

## Abstract

Increasingly stringent regulations aimed at protection of the natural environment have stimulated the search for new synthetic methodologies in organic and medicinal chemistry having no or minimum harmful effect. An interesting approach is the use of alternative activation factors, microwaves (MW) or ultrasounds (US) and also their cross-combination, which has been tested in the fast and efficient creation of new structures. At present, an easy and green hybrid strategy (“Lego” chemistry) is generally recommended for the design of new substances from different chemistry building blocks. Often, selected biologically active components with specific chemical reactivities are integrated by a suitably designed homo- or heterodifunctional linker that modifies the functionality of the starting structure, allowing easy covalent linkage to another molecule. In this study, a fast introduction of heterodifunctional halogenoacidic linker to selected mono-, di- and triphenolic active substances, allowing their functionalization, was investigated. Nucleophilic substitution reaction was chosen to produce final ethers with the reactive carboxylic group from phenols. The functionalization was performed using various green factors initiating and supporting the chemical reactions (MW, US, MW-US). The benefits of the three green supporting methods and different conditions of reactions were analyzed and compared with the results of the reaction performed by conventional methods.

## 1. Introduction

**Figure 1:**
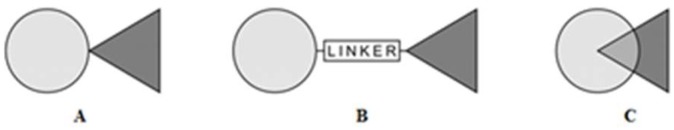
Design strategy for hybrid compounds. (**A**) Direct no-linker mode, (**B**) intermediate linker mode; (**C**) overlap mode.

The concept of green chemistry, referring to processes not harmful to the natural environment, was first used in a scientific publication in 1990 [1,2], and in 1998, Anastas and Warner [3] systematized and published 12 principles of green chemistry. These principles describe the methods of implementation of green ideas (prevent waste, atom economy, design for energy efficiency, chemical engineering, less hazardous synthesis, design of safer chemicals, design for degradation, safer solvents and auxiliaries, renewable feedstock, catalysis, reduce derivatives, pollution and accident prevention). They contain guidelines for the interdisciplinary pursuit of economical and prudent management of chemical and energy raw materials, using non-classical synthesis methods based on alternative reaction media such as water, supercritical fluids or ionic liquids. Alternative benign organic methodologies have become an imperative part of organic syntheses and chemical reactions. Various new and innovative sustainable organic reactions and methodologies using no solvents or catalysts and employing alternative energy inputs, such as microwaves, sonication, conventional and room-temperature heating conditions, and mechanochemical mixing, have been discussed [4,5]. Within the concept of green chemistry, completely solvent-free methods that eliminate the use of organic solvents have also been proposed. Solid-state reactions proceed efficiently and have many advantages (reduced pollution, low costs, simplicity of process and handling) [6,7,8]. Moreover, much attention has been paid to the use of effective and safe factors in initiating chemical reactions in modern medicinal chemistry, such as microwave irradiation (MW) [9], ultrasound (US) [10], and their cross-combinations [11,12,13,14,15]. Environmentally benign and pharmaceutically important and significant organic reactions were discussed in [4]. Novel “on-water” MW-assisted methods without any catalyst have been described [16,17,18]. Various active and natural compounds, including pharmaceutical, cosmetic and food ingredients, are reacted under mild conditions with good results [19,20]. Also, ultrasound-assisted extraction (UAE) and microwave-assisted extraction (MAE) are recognized as efficient extraction techniques that dramatically cut down working times, increasing yields and often the quality of the extract [21]. Moreover, the “Lego” chemistry concept [22,23] stimulated an interesting approach to the synthesis of innovative drug-like molecules that can accelerate the drug discovery process by utilizing a few practical and reliable reactions. The “Lego” chemistry term describes reactions that are based on readily available starting materials and reagents (chemistry building blocks), need no solvent or soft solvent (such as water), are high yielding, wide in scope, often are stereospecific, simple to perform and give a simple product easy to isolate. “Lego” and green concepts can be additionally supported by a natural additional synergy concept. The phenomenon of synergy, understood as the interaction of various factors, methods or substances, whose effect is greater than the sum of separate individual activities, is applicable in various fields. In this paper, synthetic synergy effects can be considered on two levels:

- Synergism by synthesis process intensification by means of combining two non-conventional factors: microwaves and ultrasound. These two effects of process intensification have been used to great effect in various chemical processes and engineering applications. Microwaves and ultrasounds are significantly different in terms of their nature; however, simultaneously used, they form the green SMUI technology (Simultaneous Microwave and Ultrasound Irradiation), allowing significant optimization of the chemical reaction parameters. The combination of microwaves and ultrasound has become a real chance for effective, economical and, above all, green synthetic procedures [11,12,13,14,15]. The favorable results are due to the combination of the phenomena of both factors—unique microwave heating and the phenomenon of cavitation. The microwave medium is usually combined with effective heating, and the sonification factor with efficient temperature stimulation of the chemical process. Thanks to the use of effective microwave heating, there is no loss of energy associated with conventional heating and thermal pollution of the environment is avoided. Acoustic cavitation allows thorough mixing of substrates, including two-phase mixtures, limiting the use of surfactants that have a negative impact on the environment. Thanks to cavitation, it is possible to increase the contact between the liquid substrate and the low solubility due to its fragmentation, which has a positive effect on the reaction results [11,12,13,14,15].
- Synergism by combining various active chemistry building blocks—pharmacomodulation of two biologically active structures by chemical hybridization methods leads to a new combined structure with interesting biological activity. According to the recent literature, the compounds derived from different bioactive molecules are often characterized by a synergy of their individual component activities. Many trends have been proposed for the design of new drugs containing different structures (dimers, heterodimers, heteromers, adducts, associates, complexes, biooligomers, dendrimers, dual-, bivalent-, multifunction drugs and codrugs, identical or non-identical twin drugs, mixed or combo drugs, supramolecular particles and various nanoindividuals). Chemical association of two (or more) structures into one new molecule depends on their functionality, cross-reactivity, and can be implemented by way of various methods [23,24]. Generally, the most popular molecules integration modes are: direct no-linker mode (fused hybrids), intermediate linker mode and overlap mode (merged hybrids) (Figure 1).

## 2. Results and Discussion

**Figure 2:**
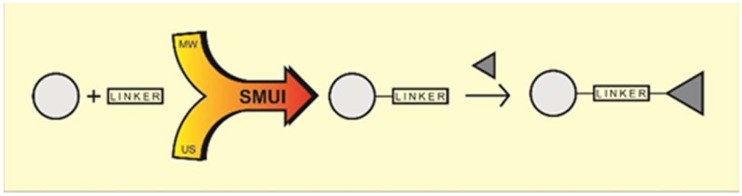
General scheme of linked hybrid derivatives synthesis.

In this paper, introduction of small heterodifunctional linkers to different phenolic molecules will be realized according to a green and synergetic synthetic strategy. Microwave heating and ultrasonic waves are among the most simple, inexpensive, and valuable tools in applied medicinal chemistry. Besides saving energy, these green techniques promote faster and more selective transformations and novel sustainable, high-throughput methods for novel drug synthesis. In perspective, this method was used to synthesize various linked hybrid derivatives from two different pharmacophores by using the linker-mode method (Figure 2).

**Figure 3:**
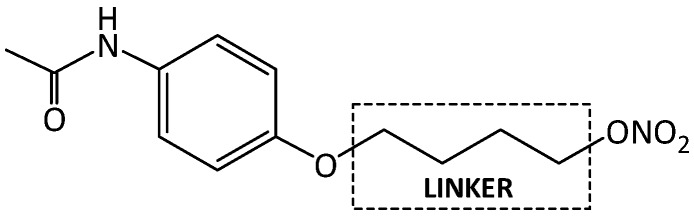
Linked hybrid nitrate derivative of paracetamol (NO-paracetamol).

Compounds containing phenolic group are commonly known and used in the pharmaceutical industry. They include salicylic acid, paracetamol, levodopa, dopamine, fenoterol and many others. Numerous hybrid combinations of classic phenolic drugs with other structures are known. One of them is a hybrid-type nitrate derivative of paracetamol [25]. Combinations of paracetamol and nitric oxide donors connected by properly selected linkers have led to the development of new compounds with promising therapeutic activities. NO-paracetamol, presented in Figure 3, is a linked-type hybrid derivative of paracetamol, which is less hepatotoxic than the parent compound in animal models. Homologous series of alkoxy acetanilides (p-methoxy, p-ethoxy: phenacetin, p-propoxy and p-butoxy acetanilides) have been shown to finally release paracetamol [26]. One of the main objectives in pharmaceutical research is to develop novel compounds showing better activity than the parent drug and less toxicity in biochemical and histopathological studies. This research also involves design of new fast and easy methods for synthesis of new compounds or modification of already known ones.

**Scheme 1:**
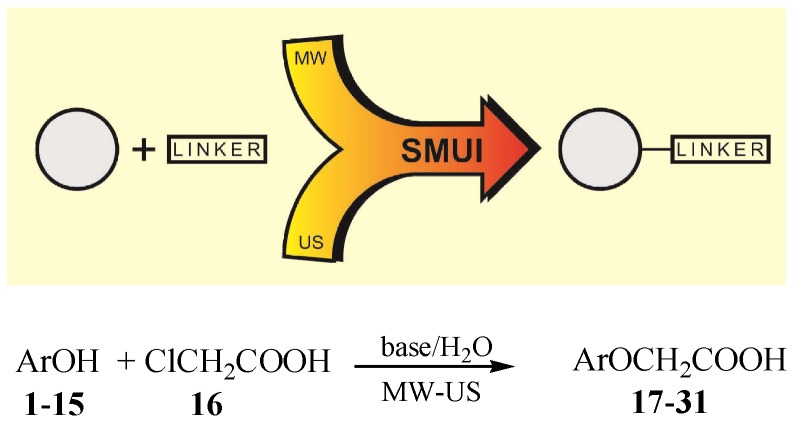
General reaction of a linker (2-chloroacetic acid) introduction to the phenol molecule; Ar: selected phenolic rest (Table 1).

In this study, the introduction of heterodifunctional halogenoacidic linker (2-chloroacetic acid) to selected phenolic substances was realized according to the alkylation reaction presented in Scheme 1. Phenoxyacetic acid derivatives with at least one reactive carboxyl group were obtained. The obtained functionalized ether derivatives of selected phenols can be successfully used in further transformations with other active molecules in accordance with the hybrid strategy (Figure 2).

Phenols can be classified, according to the number of phenolic groups, as monophenols, diphenols and triphenols (Table 1):

- monophenols: 5-methyl-2-(propan-2-yl)phenol (thymol) (**1**), 2-methoxy-4-(prop-2-enyl)phenol (eugenol) (**2**), 2-hydroxybenzoic acid (salicylic acid) (**3**), 4-hydroxybenzoic acid (**4**), its methyl ester-nipagin M (**5**) and propyl ester-nipagin P (**6**), 4-acetaminophenol (paracetamol) (**7**), 1-naphtol (**8**) and 2-naphtol (**9**);
- diphenols: 1,2-dihydroxybenzene (hydroquinone) (**10**), 1,3-dihydroxybenzene (resorcinol) (**11**), 1,7-bis(4-hydroxy-3-methoxyphenyl)hepta-1,6-dien-3,5-dione (curcumin) (**12**);
- triphenols: 1,3,5-trihydroxybenzene (phloroglucinol) (**13**), 1,2,3-trihydroxybenzene (pyrogallol) (**14**), genistein (5,7-dihydroxy-3-(4-hydroxyphenyl)chromen-4-one) (**15**).

The known biologically active structures include numerous phenolic individuals. In the present study, a variety of phenols were selected for the reaction with chloroacetic acid. Simple monophenols (**1**,**2**), diphenols (**10**,**11**) and triphenols (**13**,**14**), as well as phenols containing other functional groups, for example carboxylic groups (**3**,**4**), esters (**5**,**6**) or amide (**7**) moieties. Two naphthols (**8**,**9**) were also used in the reaction. The use of structurally diverse phenols was expected to provide information on the overall reactivity of such structures. Realization of the O-alkylation process using simple phenolic compounds allowed the application of already-tested optimal reaction conditions to selected reactions with pharmacologically active phenols. Among them, we can distinguish nipagins (**5**,**6**), showing antimicrobial action, and acetamidophenol (**7**) from the group of analgesics and antipyretics. However, the most important phenols of complex structure and not always typical reactivity are curcumin (**12**) and genistein (**15**). These compounds are particularly interesting because of the wide spectrum of their potential pharmacological activities. A fast and simple method of functionalization for these structures will open up wide possibilities of chemical hybridization towards the creation of totally novel structures with potentially interesting activity.

**Figure 4:**
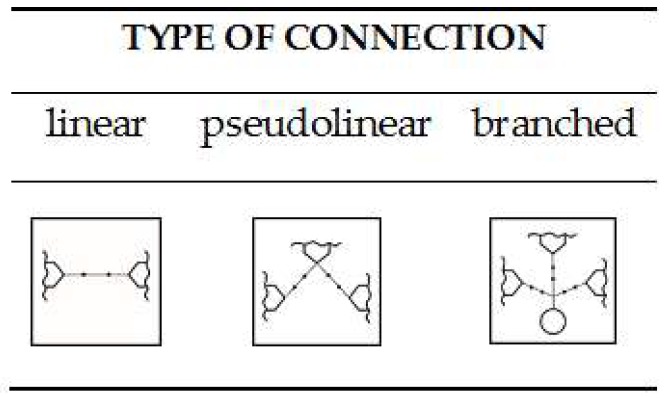
Schematic type of linked connections.

The reactions performed with selected monophenols allowed development of a general procedure for the reaction of linker insertion. However, the use of appropriate diphenols and then triphenols was not accidental, because the functionalization of these molecules using a suitably designed connector makes them potential central molecules, the so-called cores, which can be used in further research for the construction of higher molecular compounds of a certain structure, e.g., linear (derivatives of 1,4-dihydroxybenzene), pseudolinear (derivatives of 1,3-dihydroxybenzene) or branched in the case of trifunctional compounds (trihydroxybenzene derivatives), as schematically depicted in Figure 4.

The reactions of introduction of chloroacetic acid to the selected phenols [27,28] led to the formation of their mono-, di- and trisubstituted ether derivatives, respectively, according to Scheme 1. At the initial stage of research, O-alkylation reactions of the selected phenols using chloroacetic acid were carried out under the conditions of classical organic synthesis [29]. However, not all selected phenols were susceptible to reaction under the conditions used. Initially, the reactions were carried out in different solvents such as acetone, DMF (dimethylformamide), ethanol, THF (tetrahydrofuran) and water. The alkaline reaction medium was obtained using various bases such as sodium hydroxide (NaOH), potassium carbonate (K_2_CO_3_)—especially for the substrates containing a sensitive ester group, e.g., nipagins. The processes were carried out both at room temperature and at elevated temperature. Other reaction parameters were also controlled (molar ratio, type of environment, with particular emphasis on polar solvents). However, none of the classical processes allowed a satisfactory substrate conversion, especially for biologically active phenols. Clear synthetic success was obtained by using microwave and microwave-ultrasonic techniques to support chemical reactions. Recently, the impact of chemical products on the natural environment has become a very important factor that has to be considered in the design of new synthetic methodologies in organic and medicinal chemistry.

The use of alternative activation factors, microwaves (MW) or ultrasounds (US) and their mutual cross-combination has become very promising and desirable in synthetic methodologies for efficient and fast creation of drug-like structures. Microwave heating and ultrasonic waves are among the simplest, most inexpensive and valuable tools in applied chemistry. Besides saving energy, these green techniques promote faster and more selective transformations. Examples presented in the literature [11,12,13,14,15] clearly show that combined ultrasound (US) and microwave irradiation (MW), whose application is a practically hazard-free technological innovation, deserves widespread attention in fine-chemical and pharmaceutical research. Although the mechanisms of cavitation and microwave effects are not fully understood, the processes requiring enhanced heat transfer and mass transport will benefit from these green techniques [30]. Combinations of both energies may be simultaneous or sequential and conditions can be tailored for the preparative task. The results of the O-alkylation reaction conducted in this work under different reaction conditions confirmed the literature trends and the best results obtained have been compiled and presented in Table 2.

**Figure 5:**
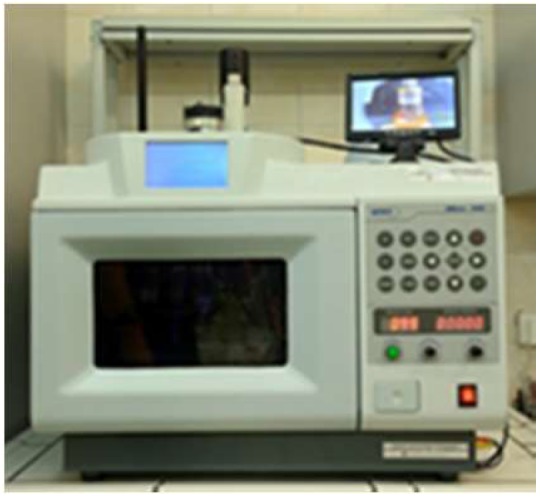
Sineo UWave-1000 multifunctional reactor.

The dedicated non-traditional Sineo reactor UWave-1000 (Figure 5) was used to carry out planned green reactions. The multifunctional chemical reactor can randomly combine, overlap and regulate microwave energy, ultraviolet light and ultrasonic waves. The SINEO reactor is equipped with a microwave generator (0–1000 W), an immersed ultrasonic working probe (26–28 kHz, 0–800W), a UV lamp (λ = 365 nm, 300 W), contact and non-contact thermometer, magnetic stirrer, cooler and reaction visualization system [31]. Almost all phenols selected were fully functionalized with 2-chloroacetic acid (**16**). Only the two phenols of key importance for pharmacological applications, i.e., curcumin (**12**) and genistein (**15**), showed partial reactivity. Despite the presence of more than one phenolic group in their molecules, monoether derivatives were only obtained by the reaction with chloroacetic acid under the conditions used. This is partly known from references [32,33,34].

Curcumin **12**, a diphenol which is composed of two feruloyl fragment connected by a carbon atom, has been a subject of many studies [32]. The expected disubstituted product could not be isolated from the reaction medium, but the presence of monosubstituted derivative **28** was detected. Review of literature [33] and experimental data do not fully explain the possibility of creating mono- or/and disubstituted curcumin derivatives. It has been suggested that the appropriate heterocyclic pyrazole and isoxazole derivatives of curcumin with blocking of the tautomeric 1,3-dicarboxylic system will lead to disubstituted derivatives. Using the MW-US method the pyrazole and isoxazole derivatives of curcumin were obtained in a much shorter time (10–20 min) than in classical conditions with a good yield [35]. In our experiment, starting from unblocked curcumin, only monosubstituted derivatives were obtained.

Another particularly interesting phenolic compound from the flavonoid group with specific reactivity is genistein **15**. It is easily alkylated using alkyl halides leading to mono-, di- and trialkyl derivatives. As a result of the reaction with chloroacetic acid **16**, only the monosubstituted ether derivative **31** is formed. However, under standard conditions, genistein is easily reacted with halogenoacetic acid ethyl ester in acetone and a catalytic amount of iodine or potassium iodide, leading to the corresponding monosubstituted ether derivative with an ester group [34]. Under the above classical conditions, the product of direct reaction of genistein **15** and free halogenoacetic acid was not obtained. The corresponding genistein carboxylic acid derivative was obtained by reacting it with a halogenoacetic acid ester and then by hydrolysis of the ester function to the free acidic group. The use of unconventional MW-US factors supporting the chemical reaction has been successful in achieving the planned compound in a direct way. The only derivative obtained was 5-hydroxy-3-(4-hydroxyphenyl)-4-oxo-4H-chromen-7-yloxy) acetic acid (**31**), similarly to in other literature reports [34]. Nipagins, i.e., the esters of p-hydroxybenzoic acid (**5** and **6**) are monophenols, which in the reaction with chloroacetic acid give their ether derivatives **20** and **21**. In the case of phenols, which also have an ester group, under NaOH basic reaction conditions, hydrolysis of the ester group was observed. Therefore, the result of the reaction of nipagins with chloroacetic acid in the presence of sodium hydroxide leads to the ethereal derivative as the main product and also p-hydroxybenzoic acid and its etherification products. Nipagins are reacted with chloroacetic acid, whereas the presence of by-products means that the separation of the neat main product becomes labor-intensive and chromatographic separation needs to be carried out. Therefore, in the case of compounds with additional sensitive functional groups, it is advisable to use milder alkaline agents, such as potassium carbonate, which will minimize the unwanted hydrolysis reaction. This O-alkylation preferably takes place towards the desired product in the presence of potassium carbonate.

Table 3 presents the initial data for the etherification reactions of one selected phenol under various conditions. At first, acetaminophenol (**7**) was reacted under standard conditions using various solvents such as water, acetone and DMF. These preliminary reactions at different temperature profiles and in different conditions did not lead to satisfactory yields. Based on the example of the results of the O-alkylation reaction, the application of MW-US method was found to be the most effective, followed by microwave and ultrasonic supporting methods applied separately. Both microwave and ultrasonic factors promoted the etherification reaction, but the simultaneous use of microwaves and ultrasound waves proved to be far more effective in reducing the reaction time and increase the chemical efficiency of the final product. In all reactions, identical molar ratios of reactants were used, corresponding to the appropriate reaction equation. In all reactions, a comparable amount of solvent was used, water in most cases. The reagents, in most cases, were soluble in the reaction medium, as they occur in the form of phenolates. However, some phenols, due to their hydrophilicity, also dissolve in water. The above homofunctional phenols are stoichiometrically reacted with a heterodifunctional linker. The molar ratios of the main reactants and additional sodium hydroxide are shown below:

- monophenol: linker: NaOH (1:1:2)
- diphenol: linker: NaOH (1:2:3)
- triphenol: linker: NaOH (1:3:4)

In the microwave-assisted process, two different reactions took place, both in aqueous solution and in solvent-free conditions. Comparing the results of MW assisted reactions, slightly better results were obtained under solvent free conditions. Elimination of solvent, especially if it is not water, in favor of a solid support is very beneficial for green reactions. Unfortunately, the elimination of solvent in the ultrasonic wave supported processes is impossible for technical reasons, as the ultrasound generating probe needs to be immersed in solution.

The reaction temperature in the MW- and MW-US-assisted reactions was higher than in the reaction under US conditions. In ultrasonic conditions, the maximum temperature was 60–70 °C. In the first two variants temperature achieved the boiling point of the solvent, i.e., water. Detailed reactor parameters and reactions are presented in Table 4.

## 3. Materials and Methods

### 3.1. Equipment and General Procedures

The melting points of all compounds used in this study were determined in open capillary tube on a Boetius apparatus and were uncorrected. MS spectra were recorded on a 402 AMD INTECTRA apparatus (AMD Intectra GmbH, Harpstedt, Germany) by the electron impact technique (EI), operating at 75 eV and ESI-MS (QTOF mass spectrometer—Impact HD, Bruker, positive ion mode). The ^1^H- and ^13^C-NMR spectra were recorded using a Varian Gemini 300 VT spectrometer, 300 and 75 MHz respectively (Agilent Technologies, Santa Clara, CA, USA). Chemical shifts (δ) were expressed in parts per million (ppm), relative to tetramethylsilane (TMS) as an internal standard, using CDCl_3_ as solvent. Coupling constants (J) are expressed in Hertz (Hz). Splitting patterns are designated as follows: s, singlet; d, doublet and m, multiplet. The progress of reactions and purity of products were checked using the TLC method on silica gel plates (25DC-Alufolien Kieselgel 60 F254 from Merck, Darmstadt, Germany). The chloroform:methanol mixtures (9:1 *v/v* and 5:1*v/v*) were used as eluents. The TLC spots on the developed plates were observed in UV light (λ = 254 nm). Silica gel 60 (63–200 μm particle size, Merck) was used for column chromatography. Phenolic compounds (paracetamol, eugenol, thymol, salicylic acid, nipain M and P, hydroquinone, resorcinol, pyrrogalol, phloroglucynol, 1- and 2-naphtol, curcumin, genistein), and solvents (DCM, methanol) from Aldrich^®^, Fluka^®^, Chempur^®^ and POCh^®^ were used. Reactions using ultrasounds, microwaves and their combination were carried out in non-traditional chemical reactor UWave-1000 (SINEO), equipped with a microwave generator (0–1000 W), an ultrasonic probe (26–28 kHz, 0–800 W), a UV lamp (λ = 365 nm, 300 W), contact and non-contact thermometer, magnetic stirrer, cooler and reaction visualization system. Flash column chromatography or crystallization process were used for final products purification. The identity of the compounds obtained was confirmed by physicochemical method and by MS spectrometry. The physicochemical and spectral data of all compounds obtained (**17**–**20**, **23**–**27**, **29**–**31**) have been confirmed by literature data [34,36,37,38,39,40,41,42,43,44,45]. The identities of compounds **17**, **21**–**23** and **28** were confirmed by additional spectral analysis.

### 3.2. Synthetic Procedures—General Procedures for Synthesis of Functionalized Phenols *(**17**–**31**)*

#### 3.2.1. US Method

In a 50 mL flask, 1 mmol of phenol (**1**–**15**), the appropriate amount of chloroacetic acid (**16**) and sodium hydroxide (potassium carbonate for nipagins) and 5mL of water were mixed.

Number of moles of chloroacetic acid (**16**) and NaOH (K_2_CO_3_):

- 1.2 mmol (0.11 g) of **16** and 2.3 mmol of base for monophenols (**1**–**9**)
- 2.2 mmol (0.21 g) of **16** and 3.3 mmol of base for diphenols (**10**–**12**)
- 3.2 mmol (0.30 g) of **16** and 4.3 mmol of base for triphenols (**13**–**15**)

A flask with carefully mixed reagents was placed in a MW-US reactor and the contents reacted under US conditions characterized by the following parameters: US = 800 W at 60–70 °C for 30 min. After the process was complete, concentrated hydrochloric acid was added to the reaction mixture until an acidic pH~3 was obtained, to isolate the free products. The separated precipitate was filtered off. The main product was purified by crystallization or column chromatography using a mixture of chloroform and methanol (9:1 or 5:1) as an eluent.

#### 3.2.2. MW Method

In a 50 mL flask, 1 mmol of phenol (**1**–**15**), the appropriate amount of chloroacetic acid (**16**) and sodium hydroxide (potassium carbonate for nipagins) were mixed.

Number of moles of chloroacetic acid (**16**) and NaOH (K_2_CO_3_):

- 1.2 mmol (0.11 g) of **16** and 2.3 mmol of base for monophenols (**1**–**9**)
- 2.2 mmol (0.21 g) of **16** and 3.3 mmol of base for diphenols (**10**–**12**)
- 3.2 mmol (0.30 g) of **16** and 4.3 mmol of base for triphenols (**13**–**15**)

A flask with carefully mixed solid reagents was placed in a MW-US reactor and the contents reacted under MW conditions characterized by the following parameters: MW = 200 W at 95–100 °C for 10min. After the process was complete, 5 mL of water was added to dissolve of solids and concentrated hydrochloric acid was added to the reaction mixture until an acidic pH~3 was obtained, to isolate the free products. The separated precipitate was filtered off. The main product was purified by crystallization or column chromatography using a mixture of chloroform and methanol (9:1 or 5:1) as an eluent.

#### 3.2.3. MW-US Method

In a 50 mL flask, 1mmol of phenol (**1**–**15**), the appropriate amount of chloroacetic acid (**16**) and sodium hydroxide (potassium carbonate for nipagins) and 5 mL of water were mixed.

Number of moles of chloroacetic acid (**16**) and NaOH (K_2_CO_3_):

- 1.2 mmol (0.11 g) of **16** and 2.3 mmol of base for monophenols (**1**–**9**)
- 2.2 mmol (0.21 g) of **16** and 3.3 mmol of base for diphenols (**10**–**12**)
- 3.2 mmol (0.30 g) of **16** and 4.3 mmol of base for triphenols (**13**–**15**)

A flask with carefully mixed reagents was placed in a MW-US reactor and the contents reacted under SMUI conditions characterized by the following parameters: MW = 200 W, US = 800 W at 95–100 °C for 10 min. After the process was complete, concentrated hydrochloric acid was added to the reaction mixture until an acidic pH~3 was obtained, to isolate the free products. The separated precipitate was filtered off. The main product was purified by crystallization or column chromatography using a mixture of chloroform and methanol (9:1 or 5:1) as an eluent.

#### 3.2.4. Classical Method

In a 50 mL flask, 1 mmol of phenol (**1**–**15**), the appropriate amount of chloroacetic acid (**16**) and sodium hydroxide (potassium carbonate for nipagins) and 5 mL of water were mixed.

Number of moles of NaOH (K_2_CO_3_):

- 1.2 mmol (0.11 g) of **16** and 2.3 mmol of base for monophenols (**1**–**9**)
- 2.2 mmol (0.21 g) of **16** and 3.3 mmol of base for diphenols (**10**–**12**)
- 3.2 mmol (0.30 g) of **16** and 4.3 mmol of base for triphenols (**13**–**15**)

A flask with mixed reagents was placed under condenser and refluxed for 7 h. After the process was complete, concentrated hydrochloric acid was added to the reaction mixture until an acidic pH~3 was obtained, to isolate the free products. The separated precipitate was filtered off. The main product was purified by crystallization or column chromatography using a mixture of chloroform and methanol (9:1 or 5:1) as an eluent.

### 3.3. The Data of Compounds *(**17**–**31**)* Obtained by MS-US Method

*2-(5-Methyl-2-propan-2-ylphenoxy)acetic acid* (**17**) [36]: in the reaction of **1** and **16** the product **17**, after crystallization process, was obtained as a light-yellow fine crystals (89%); m.p. 142–144 °C. R_f sub_ = 0.71; R_f prod_ = 0.10 (CHCl_3_: MeOH 9:1); MS, *m*/*z* (%): 208.2 (69.3) M^+^; 193.2 (100.0); 146.2 (16.4). ^1^H-NMR (CDCl_3_, δ[ppm]): 9.53 (s, 1H); 7.14–7.12 (m, 1H, Ar); 6.80–6.78 (m, 1H, Ar); 6.55 (s, 1H, Ar); 4.64 (s, 2H, OCH_2_); 3.42-3.37 (m, 1H, -CH-); 2.30 (s, 3H, -CH_3_); 1.25 (s, 3H, -CH_3_); 1.23 (s, 3H, -CH_3_). ^13^C-NMR (CDCl3, δ[ppm]): 176.68 (COOH); 154.93 (C-1Ar); 136.18 (C-5Ar); 134.32 (C-2Ar); 126.20 (C-3Ar); 122.92 (C-4Ar); 111.97 (C-6Ar); 73.2 (-OCH_2_-); 26.59 (CH_3_); 25.85 (CH_3_); 22.79 (CH); 21.28 (CH_3_).

*2-(2-Methoxy-4-prop-2-enylphenoxy)acetic acid* (**18**) [37]: in the reaction of **2** and **16** the product **18**, after crystallization process, was obtained as a cream fine crystals (86%); m.p. 89–91 °C; R_f sub_ = 0.80; R_f prod_ = 0.22 (CHCl_3_: MeOH 9:1); MS *m*/*z* (%): 222.1 (100.0) [M^+^], 163.1 (37.7), 131.1 (15.3), 115.0 (24.9), 103.0 (28.0), 91.0 (46.3), 44.9 (52.0).

*2-(Carboxymethoxy)benzoic acid* (**19**) [38]: in the reaction of **3** and **16** the product **19**, after crystallization process, was obtained as a white fine crystals (81%); m.p. 214–216 °C; R_f sub_ = 0.45; R_f prod_ = 0 (CHCl_3_: MeOH 9:1); MS *m*/*z* (%): 196.0 (100.0) [M^+^], 179.0 (7.1), 151.0 (41.5), 138.0 (3.5), 121.0 (5.1).

*4-(Carboxymethoxy)benzoic acid* (**20**) [39]: in the reaction of **4** and **16** the product **20**, after crystallization process, was obtained as a white fine crystals (96%); m.p. 290–293 °C; R_f sub_ = 0.40; R_f prod_ = 0 (CHCl_3_: MeOH 9:1); MS *m*/*z* (%): 196.0 (100.0) [M^+^], 179.0 (5.1), 151.0 (42.5), 138.0 (6.5), 121.0 (6.1).

*4-(Carboxymethoxy)benzoic acid methyl ester* (**21**): in the reaction of **5** and **16** the product **21**, after crystallization process, was obtained as a white fine crystals (75%); m.p. 143–145 °C; R_f sub_ = 0.52; R_f prod_ = 0 (CHCl_3_: MeOH 9:1); MS *m*/*z* (%): 211.2 (0.7) [M^+^], 210.1 (47.0), 179.1 (100.0), 152.0 (23.7), 121.0 (83.5), 92.0 (14.1), 76.1 (10.7). ^1^H-NMR (CDCl_3_, δ[ppm]): 9.49 (s, 1H); 7.92 (m, 2H, 3.5-Ar); 6.93 (m, 2H, 2.6-Ar); 4.64 (s, 2H, OCH_2_); 4.23 (s, 3H, CH_3_). ^13^C-NMR (CDCl_3_, δ[ppm]): 176.70 (COOH); 153.90 (C(O)O-CH_3_); 146.93 (C-1-Ar); 131.35 (C-3.5-Ar); 124.61 (C-4-Ar); 114.22 (C-2.6-Ar); 70.38 (-OCH_2_-); 59.80 (-CH_3_).

*4-(Carboxymethoxy)benzoic acid propyl ester* (**22**): in the reaction of **6** and **16** the product **22**, after crystallization process, was obtained as a white fine crystals (68%); m.p. 87–89 °C; R_f sub_ = 0.50; R_f prod_ = 0 (CDCl_3_: MeOH 9:1); MS, *m*/*z* (%): 238.0 (22.2) [M^+^]; 196.0 (100.0); 179.1 (84.6); 151.1 (26.3). ^1^H-NMR (CDCl_3_, δ[ppm]): 9.53 (s, 1H); 8.05 (m, 2H, 3.5-Ar); 7.08 (m, 2H, 2.6-Ar); 4.68 (s, 2H, OCH_2_); 4.27 (t, 2H, *J =* 6.6 Hz, OCH_2_); 1.74 (m, 2H, CH_2_); 1.24 (m, 3H, CH_3_), ^13^C-NMR (CDCl_3_, δ[ppm]): 176.14 (COOH); 154.50 (C(O)O-C_3_H_7_); 146.79 (C-1-Ar); 131.01 (C-3.5-Ar); 124.44 (C-4-Ar); 114.15 (C-2.6-Ar); 70.26 (OCH_2_); 66,62 (CH_2_CH_2_CH_3_); 22.61 (CH_2_); 11.54 (CH_3_).

*2-(4-Acetamidophenoxy)acetic acid* (**23**) [40]: in the reaction of **7** and **16** the product **23**, after crystallization process, was obtained as a beige fine crystals (86%); m.p. 87–90 °C; R_f sub_ = 0.38; R_f prod_ = 0 (CHCl_3_: MeOH 9:1); MS m/z (%): 209.1 (100.0) [M^+^], 167.0 (32.0), 151.0 (1.1), 108.0 (73.0), 80.0 (7.0). ^1^H NMR (CDCl_3_): 9.87 (s, 1H); 8.31 (br s, 1H, NH); 7.38 (m, 2H, Ar); 6.61 (m, 2H, Ar); 4.74 (s, 2H, -OCH_2_-); 2.03 (s, 3H, CH_3_). ^13^C NMR (CDCl_3_): 178.52 (COOH); 167.44 (C(O)NH); 153.15 (C-1Ar); 130.99 (C-4Ar); 120.99 (C-3.5Ar); 114.97 (C-2.6Ar); 70.43 (-OCH_2_-); 23.68 (CH_3_).

*2-Naphthalen-1-yloxyacetic acid* (**24**) [41]: in the reaction of **8** and **16** the product **24**, after crystallization process, was obtained as a blue-grey fine crystals (88%); m.p. 175–177 °C; R_f sub_ = 0.59; R_f prod_ = 0.14 (CHCl_3_: MeOH 9:1); MS *m*/*z* (%): 201.9 (89.7) [M^+^], 200.5 (78.1), 157.4 (20.0), 143.6 (100.0), 126.4 (53.8), 115.6 (91.5).

*2-Naphtalen-2-yloxyacetic acid* (**25**) [41]: in the reaction of **9** and **16** the product **25**, after crystallization process, was obtained as a light-grey fine crystals (91%); m.p. 85–87 °C; R_f sub_ = 0.40; R_f prod_ = 0.16 (CHCl_3_: MeOH 9:1); MS *m*/*z* (%): 201.9 (89.3) [M+], 200.5 (76.2), 157.5 (20.5), 143.6 (100.0), 126.4 (53.1), 115.6 (91.2).

*2-[4-(Carboxymethoxy)phenyloxy]acetic acid* (**26**) [42]: in the reaction of **10** and **16** the product **26**, after crystallization process, was obtained as a beige fine crystals (84%); m.p. 252–255 °C; R_f sub_ = 0.22; R_f prod_ = 0 (CDCl_3_: MeOH 9:1); MS *m*/*z* (%): 226.1 (42.0) [M^+^], 167.0 (31.7), 108.9 (100.0), 81.0 (13.2).

*2-[3-(Caroxymethoxy)phenoxy]acetic acid* (**27**) [43]: in the reaction of **11** and **16** the product **27**, after crystallization process, was obtained as a light-beige fine crystals (87%); m.p. 206–209 °C; R_f sub_ = 0.56; R_f prod_ = 0.22 (CHCl_3_: MeOH 5:1); MS *m*/*z* (%): 226.1 (42.0) [M^+^], 167.0 (31.7), 108.9 (100.0), 81.0 (13.2).

*2-{4-[7-(4-Hydroxy-3-metoxyphenyl)-3,5-dioxohepta-1,6-dienyl]-2-metoxyphenoxy}acetic acid* (**28**): in the reaction of **12** and **16** the product **28**, after column chromatography, was obtained as a brick red fine crystals; m.p. 153–155 °C; R_f sub_ = 0.6; R_f prod_ = 0 (CHCl_3_: MeOH 9:1); MS m/z (%): 426.3 (8.0) [M^+^], 410.2 (12.7), 368.2 (47.9), 326.1 (37.9), 177.0 (100.0), 150,0 (30,8). ^1^H-NMR (CDCl_3_,δ [ppm]): 16.03 (broad s, 1H, OH_enol_); 9.49 (s, 1H, COOH); 7.54 (d, *J* = 16 Hz, 2H, HC=C, 1.7-H); 7.11 (m, 2H, 6-Ar); 6.87 (m, 4H, 2.5-Ar); 6.49 (d, *J* = 16, 2H, =CH-CO-, 2.6-H); 5.84 (broad s, 1H, OH_phenol_); 5.79 (s, 1H, -CH=C(OH)-, 4-H); 4.64 (s, 2H, OCH_2_); 3.88 (s, 6H, 3-Ar-OCH_3_). ^13^C-NMR (CDCl_3_, δ [ppm]): 183.16 (C3); 176.65 (COOH); 147.75 (C4-Ar); 146,64 (C3-Ar); 140.32 (C1); 127.58 (C1-Ar); 122.72 (C6-Ar); 121.65 (C2); 114.57 (C5-Ar); 109.45 (C2-Ar); 101.12 (C4); 70.37 (OCH_2_); 55.88 (OCH_3_).

*2-[3,5-Bis(carboxymethoxy)phenoxy]acetic acid* (**29**) [44]: in the reaction of **13** and **16** the product **29**, after column chromatography, was obtained as a colorless resin (79%); R_f sub_ = 0.28; R_f prod_ = 0 (CDCl_3_: MeOH 5:1); MS *m*/*z* (%): 300.2 (1.2) [M], 298.1 (33.3), 252.2 (35.7), 240.2 (7.7), 224.3 (22.4), 212.2 (100.0), 178.0 (29.8), 166.0 (46.9), 138.1 (20.4).

*2-[2,3-Bis(carboxymethoxy)phenoxy]acetic acid* (**30**) [45]: in the reaction of **14** and **16** the product **30**, after crystallization process, was obtained as a light-beige fine crystals (83%); m.p. 317–319 °C; R_f sub_ = 0.37; R_f prod_ = 0 (CDCl_3_: MeOH 5:1); MS *m*/*z* (%): 300.1 (33.8) [M^+^], 242.1 (5.2), 224.0 (9.3), 183.0 (21.2), 151.0 (41.2), 136.9 (100.0), 124.9 (24.5), 106.9 (46.0), 94.9 (30.2), 79.0 (16.7).

*2-[5-Hydroxy-3-(4-hydroxyphenyl)-4-oxo-4H-chromen-7-yloxy)] acetic acid* (**31**) [34]: in the reaction of **15** and **16** the product **31**, after column chromatography, was obtained as a light-brown fine crystals (41%); m.p. 257–259 °C; R_f sub_ = 0.45; R_f prod_ = 0 (CHCl_3_: MeOH 9:1); MS *m*/*z* (%): 328.0 (100.0) [M^+^], 283.0 (21.1), 269.0 (63.4), 241.0 (21.1), 148.9 (12.1), 123.9 (10.4), 88.8 (2.3).

## 4. Conclusions

The results of the experiments performed confirmed the beneficial effect of non-classical reaction conditions. The reactions assisted with ultrasound, microwaves and above all their combination are characterized by a unique and specific synergy of action. In this study, we analyzed the reactions of O-alkylation of selected mono-, di- and triphenols with chloroacetic acid that can be used for rapid and clean functionalization or for introducing changes in the reactivity of selected active structures, not only of phenol type. The choice of the reaction will depend on the type of active structure chosen and the chemical nature of the linker introduced to it. Microwave-ultrasonic-assisted processes can successfully compete with classical synthesis methods. Results of our study confirm the literature described tendency of growing effectiveness of reaction assistance in the sequence: US, MW, MW-US. All reactions were carried out in an aquatic environment under the influence of green initiating agents, and so had no negative impact on the environment. Phenoxyacetic acid derivatives with at least one reactive carboxyl group were obtained. The obtained functionalized ether derivatives of selected phenols can be successfully used in further transformations with other active molecules in accordance with the currently applicable hybrid strategy. The use of a rational, green and synergistic synthetic approach in obtaining new ingredients for applications in the pharmaceutical field is very promising area that perfectly fits in the actual trends in medical chemistry.

## Figures and Tables

**Table 1:** Structures of phenols selected and products obtained.

	No	SUBSTRAT	No	PRODUCT
**MONOPHENOLS**	**1**	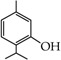	**17**	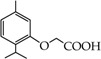
	**2**	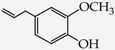	**18**	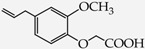
	**3**	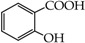	**19**	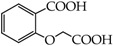
	**4**	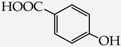	**20**	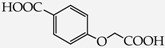
	**5**	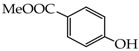	**21**	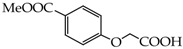
	**6**	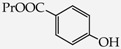	**22**	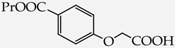
	**7**	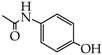	**23**	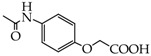
	**8**	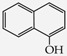	**24**	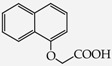
	**9**	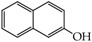	**25**	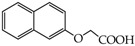
**DIPHENOLS**	**10**	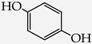	**26**	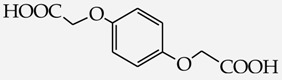
	**11**	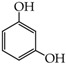	**27**	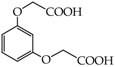
	**12**	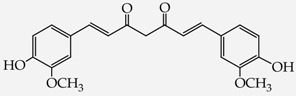	**28**	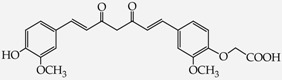
**TRIPHENOLS**	**13**	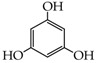	**29**	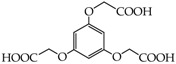
	**14**	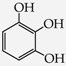	**30**	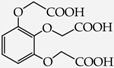
	**15**	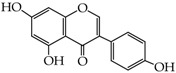	**31**	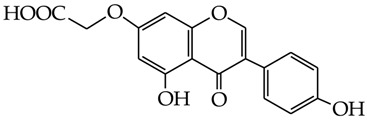

**Table 2:** The main parameters of O-alkylation reaction.

Substrat Product	Name	US ^1^		MW ^2^		MW-US ^3^		Classic ^4^	
		Time min	%	Time min	%	Time min	%	Time h	%
**1**	5-methyl-2-(propan-2-yl)phenol (thymol)	30	40	10	95	5	89	8	19
**17**	2-[5-methyl-2-(propan-2-yl)phenoxy]acetic acid								
**2**	2-methoxy-4-(prop-2-enyl)phenol (eugenol)	30	45	10	92	5	86	8	21
**18**	2-[2-methoxy-4-(prop-2-enyl)phenoxy]acetic acid								
**3**	2-Hydroxybenzoic acid (salicylic acid)	30	22	10	75	7	81	-	-
**19**	2-(Carboxymethoxy)benzoic acid								
**4**	4-Hydroxybenzoic acid	30	37	10	89	5	96	-	-
**20**	4-(Carboxymethoxy)benzoic acid								
**5**	4-Hydroxybenzoic acid methyl ester (nipagin M)	30	20 *	10	58 *	5	75 *	8	25
**21**	4-(Carboxymethoxy)benzoic acid methyl ester								
**6**	4-Hydroxybenzoic acid propyl ester (nipagin P)	30	31 *	10	67 *	7	68 *	8	35
**22**	4-(Carboxymethoxy)benzoic acid propyl ester								
**7**	N-(4-hydroxyphenyl)acetamide (paracetamol)	30	27	10	68	5	86	7	23
**23**	2-(4-acetamidophenoxy)acetic acid								
**8**	1-Naphtol	30	35	10	72	10	88	8	33
**24**	2-Naphtalen-1-yloxyacetic acid								
**9**	2-Naphtol	30	38	10	75	10	91	-	-
**25**	2-Naphtalen-2-yloxyacetic acid								
**10**	1,4-Dihybroxybenzen (hydrochinon)	30	40	10	54	10	84	8	36
**26**	2-[4-(Carboxymethoxy)phenoxy]acetic acid								
**11**	1,3-Dihydroxybenzen (resorcinol)	30	32	10	64	10	87	-	-
**27**	2-[3-(Carboxymetoxy)phenoxy]acetic acid								
**12**	1,7-Bis(4-hydroxy-3-methoxyphenyl)hepta-1,6-dien-3,5-dione (curcumin)	30	-	10	15	10	36	8	-
**28**	2-{4-[7-(4-hydroxy-3-metoxyphenyl)-3,5-dioxohepta-1,6-dienyl]-2-metoxyphenoxy}acetic acid								
**13**	1,3,5-Trihydroksybenzen (phloroglucinol)	30	39	10	66	10	79	8	38
**29**	2-[3,5-(Bis-carboxymetoxy)phenoxy]acetic acid								
**14**	1,2,3-Trihydroxybezen (pyrogallol)	30	29	10	71	10	83	-	-
**30**	2-[2,3-Bis(carboxymetoxy)phenoxy]acetic acid								
**15**	5,7-Dihydroxy-3-(4-hydroxy phenyl)chromen-4-one (genistein)	30	-	10	-	30	41	8	-
**31**	2-[5-Hydroxy-3-(4-hydroxyphenyl)-4-oxo-4H-chromen-7-yloxy] acetic acid								

^1^ US-800W/60–70 °C; ^2^ MW/200W/NaOH/T~100 °C (solvent free); ^3^ MW-200W/US-100W/T~100 °C; ^4^ NaOH or K_2_CO_3_*/H_2_O/bp. * for nipagins K_2_CO_3_ was used instead of NaOH for ester group protection.

**Table 3:** Conditions of O-alkylation reaction of phenol **7** (paracetamol).

Method	Condition	Time	[%]
Classic	NaOH/H_2_O/reflux	7 h	23
Classic	DMF/K_2_CO_3_/rt	24 h	-
Classic	Aceton/K_2_CO_3_/rt	24 h	-
US	US-800W/60–70 °C	30 min	27
MW/solvent free	MW/200W/NaOH	10 min	68
MW	MW/200W/NaOH/H_2_O/~100 °C	10 min	52
MW-US	MW-200W/US-800W/~100 °C	5–10 min	86

**Table 4:** The main reaction parameters in the UWave-1000 reactor.

	US	MW	MW-US
Power	800 W	200 W	∑_MW+US_
Frequency	28 KHz	2450 MHz	∑_MW+US_
Temperature	60–70 °C	95–100 °C	95–100 °C
Time	30 min	10 min	5–10 min
